# Glutathione S-Transferase Genes Involved in Response to Short-Term Heat Stress in *Tetranychus urticae* (Koch)

**DOI:** 10.3390/antiox13040442

**Published:** 2024-04-08

**Authors:** Tong Zhu, Bin Wei, Yue Wang, Suqin Shang

**Affiliations:** Biocontrol Engineering Laboratory of Crop Diseases and Pests of Gansu Province, College of Plant Protection, Gansu Agricultural University, Lanzhou 730070, China; zhutong0213@163.com (T.Z.); gsndwb@126.com (B.W.); 18418127029@163.com (Y.W.)

**Keywords:** *Tetrarcychus urticae*, Glutathione S-transferase, heat stress, oxidative stress

## Abstract

*Tetranychus urticae*, a globally ubiquitous mite, poses a significant threat to agriculture. Elevated temperatures exacerbate the growth, development, and reproduction of *T*. *urticae*, leading to substantial crop damage. In this study, we employed comparative transcriptomic approaches with whole-genome information of *T. urticae* to identify six Glutathione S-transferase genes (GSTs) implicated in heat stress response. Through comprehensive bioinformatics analyses, we elucidated the tertiary structure and active sites of the corresponding proteins, providing a thorough characterization of these GST genes. Furthermore, we investigated the expression patterns of these six GST genes under short-term heat shock conditions. Our findings unveiled the involvement of *T. urticae* GST genes in combating oxidative stress induced by heat, underscoring their role in antioxidant defense mechanisms. This study contributes valuable insights into the molecular mechanisms underlying the response of *T. urticae* to heat stress, laying a foundation for the development of strategies aimed at mitigating its impact in high-temperature environments.

## 1. Introduction

The two-spotted spider mite, *Tetranychus urticae* (Koch), represents a pervasive global threat to agricultural productivity [1], wreaking havoc on crops, fruit trees, and vegetables [2,3,4], particularly in greenhouse and controlled environment agriculture settings [5]. These minuscule pests take up residence on the undersides of plant leaves, spinning intricate webs to create microhabitats that shield them from environmental stressors, ward off predators, enable pheromonal communication, and facilitate dispersion [6]. During the scorching summer months, natural ecosystems and controlled agricultural environments contend with soaring temperatures, which provide ideal conditions for the proliferation of *T. urticae* populations [7]. Elevated temperatures not only result in more giant adult female mites and increased egg production but also diminish the size of individual eggs [8]. The remarkable adaptability of *T. urticae* to such high-temperature stressors inflicts significant damage to crops, leading to substantial economic losses and jeopardizing food security.

In an effort to unravel the adaptive strategies employed by *T. urticae* to cope with high-temperature stress, our previous research uncovered compelling insights. We observed a marked rise in total antioxidant capacity (T-AOC) and the activity levels of three key antioxidant enzymes following short-term heat exposure in *T. urticae*. This phenomenon suggests that high temperatures induce oxidative stress in these mites, triggering an accumulation of reactive oxygen species (ROS) [9]. However, the heightened activity of antioxidant enzymes effectively scavenges these harmful ROS, bolstering *T. urticae*’s resilience to high temperatures.

Too high or too low temperature, too strong or weak light intensity, and ultraviolet radiation will cause oxidative stress in organisms [10]. When oxidative stress occurs in the body, many reactive oxygen species (ROS) (O_2_^−^, OH^−^, and H_2_O_2_) will be generated immediately [11]. ROS will increase membrane permeability, lipid peroxidation, and cell apoptosis, harming organisms [12]. Antioxidant enzymes play an essential role in coping with oxidative stress and regulating apoptosis and longevity. Superoxide dismutases (SOD), peroxidase (Prx), catalase (CAT), Glutathione peroxidase (GPX), and Glutathione S-transferase (GST) have been reported as the key antioxidant enzymes involved in coping with high-temperature stress in mites and insects [13]. ROS first reacts with SOD to produce H_2_O_2_ and O_2_ [14], and then H_2_O_2_ can combine with Prx and CAT to produce H_2_O and O_2_ on the one hand [15]. H_2_O_2_ can also combine with Glutathione (GSH) and GST to produce water and other nontoxic substances under the catalysis of GPX, on the other hand, to complete the decomposition of H_2_O_2_ [16].

GSTs are vital multifunctional proteins within organisms, wielding the power to engage in detoxification and substance metabolism [17]. They protect against exogenous pesticides [18], plant secondary substances [19], and ROS damage [20]. Typically existing as dimers, GSTs catalyze the conjugation of reduced glutathione to the electrophilic groups of endogenous or exogenous substances [21]. This catalytic process aids in the expulsion of harmful substances from the organism, fortifying it against poisoning [22]. Beyond their detoxifying prowess, GSTs also play a pivotal role in neutralizing highly oxidizing ROS such as O_2_^−^, OH^−^, and H_2_O_2_, as well as lipid peroxides like malondialdehyde (MDA) [23], thus safeguarding organisms from ROS-induced damage [24]. Although the metabolic mechanism of chemical pesticides garners significant attention in GST studies, scant research delves into the role of GSTs in conferring bioheat resistance.

The publication of the whole genome of *T. urticae* in 2011 unveiled the presence of 31 GST genes in its genetic blueprint. These include 16 Delta family GSTs, 12 Mu family GSTs, 2 Omega family GSTs, and 1 Theta family GST [25]. To unearth whether GST genes participate in *T. urticae*’s heat resistance mechanism, we subjected adult female *T. urticae* to transcriptomic sequencing following short-term high-temperature stress (with 25 °C as the negative control). Our study seamlessly integrated whole-genome and transcriptomic data, identifying six GST genes exhibiting up-regulated expression levels post short-term heat shock. The bioinformatic analysis of these genes unveiled their distinctive characteristics. Subsequently, real-time quantitative PCR (RT-qPCR) was employed to scrutinize the expression patterns of these genes in response to short-term high-temperature stress. This endeavor furnishes a foundational framework for dissecting the mechanisms underpinning *T. urticae*’s heat resistance and devising effective preventive measures against it.

## 2. Materials and Methods

### 2.1. Mite Colony

The *Tetranychus urticae* population utilized in this research originated from the Laboratory of Insect Systematic and Biodiversity at Gansu Agricultural University in Lanzhou, Gansu, China. Since 2012, these mites have been maintained without pesticide exposure or temperature extremes. They were cultivated on fresh bean plants (*Phaseolus vulgaris* L.) within controlled climate chambers set at 25 ± 1 °C, with a relative humidity of 60 ± 5%, and subjected to an L16: D8 photoperiod, all under pesticide-free conditions.

### 2.2. Selection of GST Genes

From the comprehensive data set encompassing 31 GST genes as reported in the whole genome information and the outcomes of transcriptome sequencing (Accession number: PRJNA1073827), we identified GST genes exhibiting up-regulated expression levels through the analysis of differential expressed genes (DEGs). The Log2FC and *p*-value were calculated using the DEseq2 method, and data visualization was performed using GraphPad Prism (version 8.0). 

### 2.3. Cloning the CDSs of GST Genes

The coding sequences (CDSs) of GST genes were determined utilizing Open Reading Frame Finder (ORF) (https://www.ncbi.nlm.nih.gov/orffinder/ (accessed on 3 January 2024)) in NCBI. Subsequently, primers (refer to Table S1) were designed using the Prime-blast online software (https://www.ncbi.nlm.nih.gov/tools/primer-blast/ (accessed on 3 January 2024)). Three hundred adult female mites were meticulously selected for experimental procedures, and total RNA was extracted from the samples using Trizol reagent (Takara, Dalian, China). The RNA was then reverse-transcribed into cDNA utilizing the PrimeScript RT reagent Kit (Takara, Dalian, China). The resultant first-strand cDNA was subjected to PCR amplification, and the PCR products were ligated into the pLB-T vector (TIANGEN, Beijing, China). Following this, the constructs were introduced into Top10 Escherichia coli cultures (TIANGEN, Beijing, China), and positive clones were discerned and selected for sequencing, a service provided by Tsingke Biotech Co., Ltd. (Beijing, China). The sequencing outcomes were subsequently assembled and compared utilizing DNAMAN software (version 6.0).

### 2.4. Bioinformatic Analysis and Identification of GST Genes

The molecular weights and theoretical isoelectric points were calculated using the ExPASy ProtParam tool (http://web.expasy.org/protparam// (accessed on 17 June 2023)). To construct a phylogenetic tree, we employed the maximum likelihood approach method (1000 replications) in Molecular Evolutionary Genetics Analysis (MEGA) v.11.0 (Sudhir Kumar, PA, USA, https://megasoftware.net/ (accessed on 17 June 2023)), based on the sequences of the 6 GST genes.

### 2.5. Structural Characterization of the Coding Region of 6 GST Genes

The structural analysis of GST genes was forecasted using NCBI’s online Conserved Domain Search Service (CD Search) (https://www.ncbi.nlm.nih.gov/Structure/cdd/wrpsb.cgi (accessed on 3 January 2024)). Subsequently, the tertiary structure of the six GST genes was predicted and constructed using the AlphaFold2 online tool [26]. Finally, the visual representations of these predicted structures were generated using PYMOL software (Version 2.5.7) [27].

### 2.6. Expression of GST Transcripts

In this study, we investigated the transcriptional expression of antioxidant enzyme genes via RT-qPCR. Furthermore, the α-tubulin gene (Accession number: JN881327.1) for normalization, and the primer sequences utilized are detailed in Table S2. Three hundred adult females were carefully collected with a small brush, transferred into 1.5 mL microtubes, and placed in an artificial control chamber. Samples underwent treatment at varying temperatures (36, 39, and 42 °C) for different durations (2, 4, and 6 h), all within controlled humidity conditions (RH 80 ± 5%). Following treatment, the samples were promptly frozen with liquid nitrogen and preserved at −80 °C in a refrigerator. Adult females reared at 25 °C for each treatment served as negative controls. RNA extraction from each sample was performed using Trizol reagent (Thermo Fisher Scientific, New York, NY, USA), and the quantity and quality of RNA samples were evaluated using a Thermo Scientific NanoDropTM 2000 UV-VIS Spectrophotometer (Thermo Fisher Scientific, New York, NY, USA). Each treatment involved the extraction of RNA from three hundred mites, with five replicates for each treatment. Approximately 1 μg of RNA from each sample was reverse-transcribed into cDNA using the PrimerScriptTM RT reagent Kit with gDNA Eraser (TaKaRa, Dalian, China). RT-qPCR analysis was performed using the ABI QuantStudio 5 Real-Time PCR system (Thermo Fisher Scientific, New York, NY, USA).

### 2.7. Statistical Analysis

Quantitative real-time PCR (qRT-PCR) analyses were conducted to assess gene expression levels, utilizing the relative quantification 2^−∆∆CT^ method [28]. A paired-samples *t*-test was employed to evaluate the susceptibility of *T. urticae* to short-term heat stress. A significance threshold of *p* < 0.05 was utilized to determine statistical significance.

## 3. Results

### 3.1. Selection and Identification of GST Genes in T. urticae

**Figure 1:**
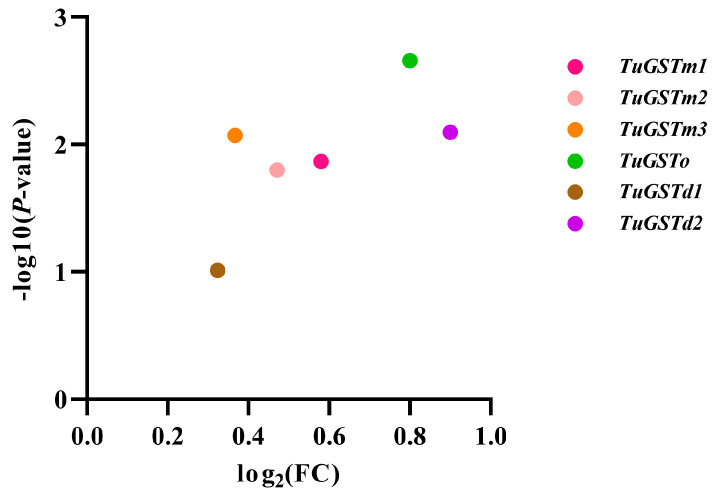
Volcanic plot of six GST genes. The final result is based on FPKM as the original data (Figure 2B) (the source data is in Table S3); the Log2FC and *p*-value were calculated using the DEseq2 method; and data visualization was performed using GraphPad Prism (version 8.0).

**Figure 2:**
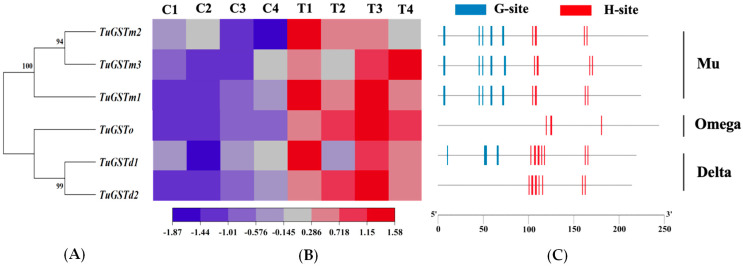
Characterization of six GST Genes in *T. urticae*. Six GST genes were identified as belonging to three subfamilies, including three Mu family GSTs, one Omega family GST, and two delta family GSTs. (**A**) Phylogenetic evolutionary analysis of GST genes. A phylogenetic tree was constructed using MEGA (Version. 11), using the maximum likelihood approach method based on 1000 replicates. (**B**) Heat map of the response of GST genes to heat stress. C1–4 represent four separate values for the control samples. T1–4 represent four separate values for the treatment samples. RSEM was used to calculate the gene expression levels of each sample. The heatmap function was used for the hierarchical clustering analysis of the six GST genes. The color scale at the bottom denotes the FPKM value from the lowest (blue) to the highest (red). (**C**) Structural analysis of GST genes. The structural analysis of the GST genes was predicted using NCBI online software Conserved Domain Search Service (CD Search) and visualized using TBtools software. The small vertical icons indicate the amino acids involved in binding sites. The blue icons represent the GSH-binding site of the GSTs (G-site), and the red icons represent the substrate-binding site of the GSTs (H-site).

Based on the comprehensive analysis incorporating whole genome information of *T. urticae* and transcriptional data following short-term heat shock (39 °C–4 h) compared to a control group (25 °C), we identified six GST genes that exhibited upregulation post short-term heat stress. These genes were thoroughly annotated in NCBI and designated as *TuGSTm1*, *TuGSTm2*, *TuGSTm3*, *TuGSTo*, *TuGSTd1*, and *TuGSTd2* (Gene entry numbers: XM_015925628.2, XM_015927346.2, XM_015927509.2, XM_015932051.2, XM_015936066.2, and XM_015937313.2) (see Figure 1). Notably, the FPKM (fragments per kilobase of transcript per million mapped reads) values of all six genes significantly increased following short-term heat shock (refer to Figure 2B). Furthermore, upon cloning the coding sequence of these genes, our results demonstrated complete consistency with the sequences archived in NCBI, with no observed base or amino acid mutations. This consistency underscores the reliability and accuracy of our findings.

### 3.2. Sequence Analysis of Six GST Genes

The characteristics of the six GST genes are summarized in Table 1. The CDS sequence lengths (Open reading frame, ORF) ranged from 642 bp to 732 bp, encoding amino acid (aa) sequences from 192 to 244 residues. The corresponding molecular sizes varied between 22.447 and 27.957 kDa, with theoretical isoelectric points ranging from 4.71 to 6.12. The molecular formulas for each gene are presented in Table 1.

Phylogenetic analysis revealed that the six genes clustered into three distinct GST families: three Mu family GSTs, one Omega family GST, and two Delta family GSTs (see Figure 2A). Notably, TuGSTm2 and TuGSTm3 exhibited the closest evolutionary relationship, while TuGSTd1 and TuGSTd2 also showed a close evolutionary association. This phylogenetic arrangement provides insights into the evolutionary relationships among the GST genes under investigation.

### 3.3. Structural Characterization of Six GST Genes

Through the prediction of conserved domains, we successfully identified the glutathione (GSH)-binding sites of the GSTs, commonly referred to as G-sites, in all genes except *TuGSTo* and *TuGSTd2*. Additionally, we predicted the substrate-binding sites of the GSTs, known as H-sites, across all six genes. Based on predictive software, all G-sites were found to reside within the N-terminal domain, whereas all H-sites were situated within the C-terminal domain (refer to Figure 2C). This distribution pattern sheds light on the structural organization of GST proteins and underscores the functional significance of distinct domains in substrate and cofactor binding.

**Figure 3:**
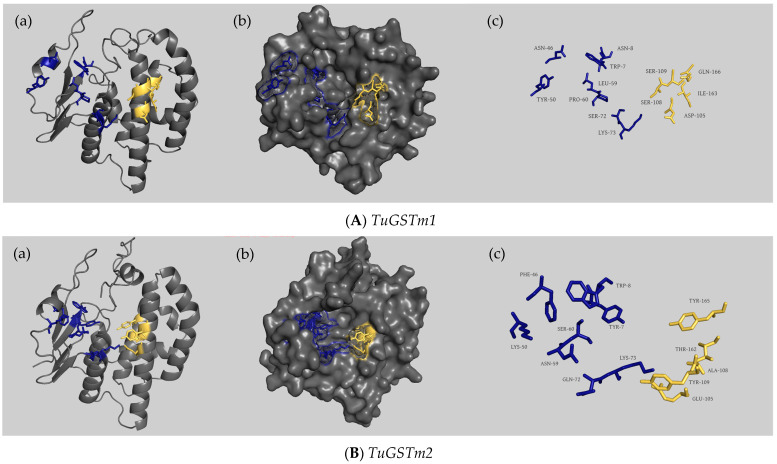

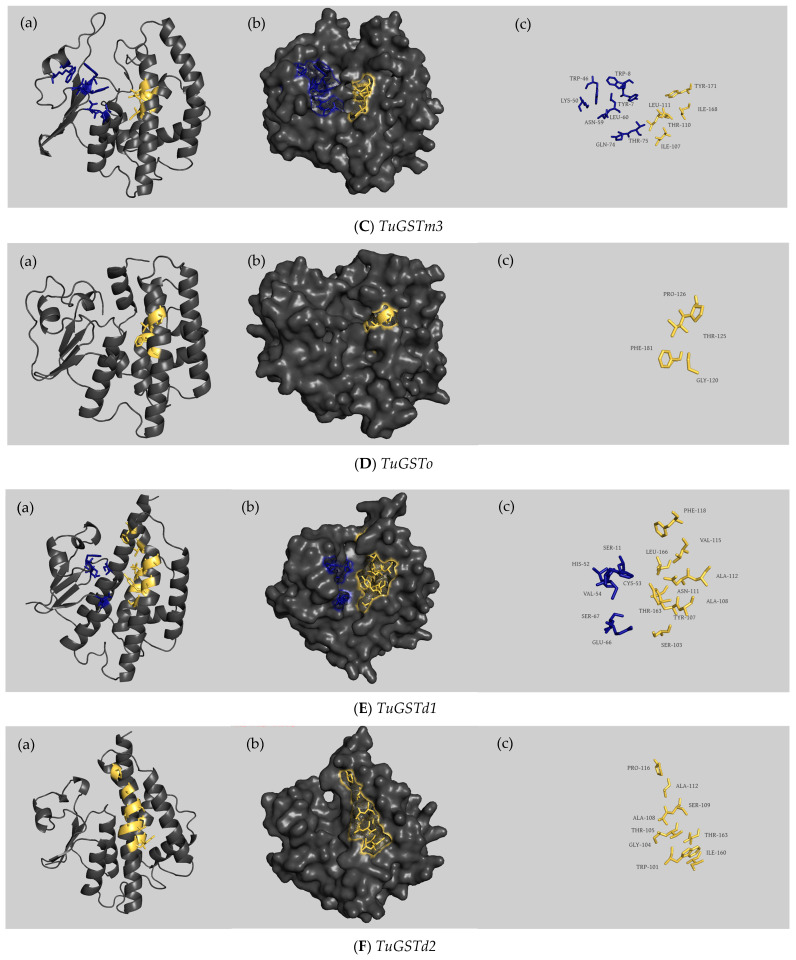
The overall predicted structure of the six GSTs ((**A**) *TuGSTm1*; (**B**) *TuGSTm2*; (**C**) *TuGSTm3*; (**D**) *TuGSTo*; (**E**) *TuGSTd1*; and (**F**) *TuGSTd2*). (a) Visualization of GST structure in cartoon; G-sites are shown with blue stick structure, and H-sites are shown with yellow stick structure. (b) Surface representation of GST genes and the active binding sites are highlighted. Active site residues that are conserved in all GSTs are highlighted in blue and yellow. The blue part indicates the GSH-binding active sites, and the yellow part indicates the substrate-binding sites. (c) All the active sites of GSTs. The amino acid stick structure at the G-sites is shown in blue, and the amino acid stick structure at the H-sites is shown in yellow. The structural model was built using AlphaFold2 and the results were visualized using PYMOL.

Each of the six genes was found to possess a single α-helical domain, and the active catalytic sites within the same family exhibited remarkable similarity (refer to Figure 2 and Figure 3). For *TuGSTm1*, there were eight G-sites (Trp^7^, Asn^8^, Asn^46^, Tyr^50^, Leu^59^, Pro^60^, Ser^72^, and Lys^73^) and five H-sites (Asp^105^, Ser^108^, Ser^109^, Ile^163^, and Gln^166^). *TuGSTm2* displayed eight G-sites (Tyr^7^, Trp^8^, Phe^46^, Lys^50^, Asn^59^, Ser^60^, Gln^72^, and Lys^73^) and five H-sites (Glu^105^, Ala^108^, Tyr^109^, Thr^162^, and Tyr^165^). In *TuGSTm3*, there were eight G-sites (Tyr^7^, Trp^8^, Trp^46^, Lys^50^, Asn^59^, Leu^60^, Gln^74^, and Thr^75^) and five H-sites (Ile^107^, Thr^110^, Leu^111^, Ile^168^, and Tyr^171^). TuGSTo exhibited four H-sites (Gly^120^, Thr^125^, Pro^126^, and Phe^181^). *TuGSTd1* showcased six G-sites (Ser^11^, His^52^, Cys^53^, Val^54^, Glu^66^, and Ser^67^) and nine H-sites (Ser^103^, Tyr^107^, Ala^108^, Asn^111^, Ala^112^, Val^115^, Phe^118^, Thr^163^, and Leu^166^). Lastly, *TuGSTd2* presented nine H-sites (Trp^101^, Gly^104^, Thr^105^, Ala^108^, Ser^109^, Ala^112^, Pro^116^, Ile^160^, and Thr^163^). It is important to note that although the G-site of *TuGSTo* and *TuGSTd2* was not directly predicted, it does not necessarily imply its absence.

### 3.4. Transcriptional Expression of Six GST Genes under Different Heat Stress Conditions

**Figure 4:**
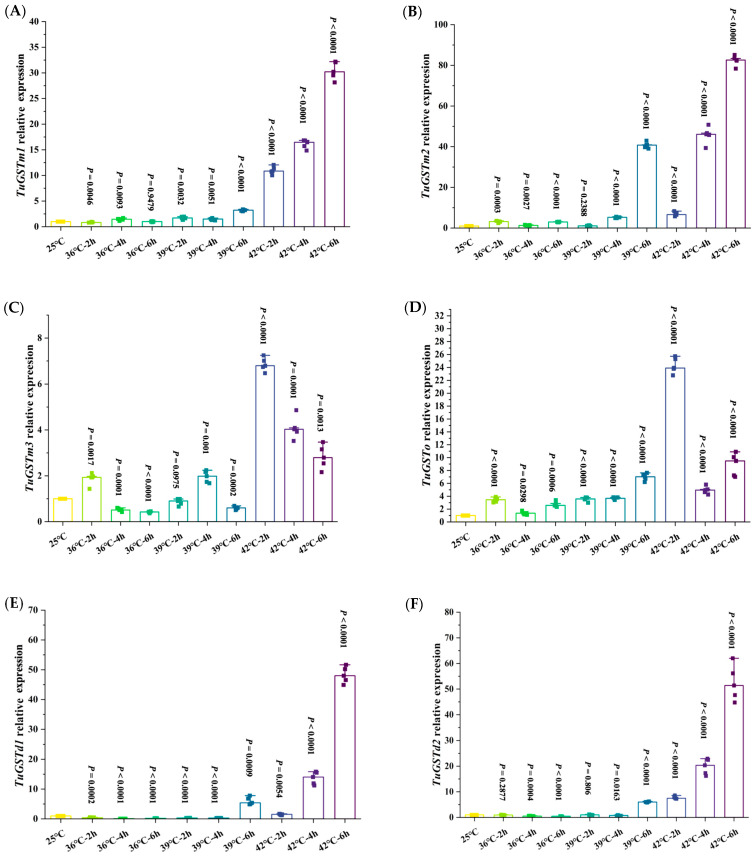
Relative expression levels of GST genes ((**A**) *TuGSTm1*, (**B**) *TuGSTm2*, (**C**) *TuGSTm3*, (**D**) *TuGSTo*, (**E**) *TuGSTd1*, and (**F**) *TuGSTd2*) in *T. urticae*. The statistical difference was determined using the paired samples *t*-test, data are median, n = 5 biologically independent samples. The measure of variation is the median. The source data is in Table S4.

When exposed to 36 °C, the relative expression level of *TuGSTm1* was significantly down-regulated after 36 °C for 2 h. With the extension of exposure time, the relative expression level of *TuGSTm1* was significantly increased and reached the maximum at 36 °C for 4 h, and then returned to the normal level at 36 °C for 6 h. When exposed to 39 °C, the relative expression levels of *TuGSTm1* were significantly up-regulated, rising first, then decreasing, and finally reaching the maximum at 39 °C for 6 h. The relative expression level of the *TuGSTm1* gene was significantly up-regulated at 42 °C and increased with the extension of exposure time, reaching the maximum at 6 h (Figure 4A).

When exposed to 36 °C, the relative expression level of *TuGSTm2* was significantly up-regulated, reaching the maximum at 36 °C for 2 h, and then showing a trend of first decreasing and then increasing. When exposed to 39 °C, the relative expression level of *TuGSTm2* was not significantly different from that of the control group after 39 °C for 2 h, but with the extension of exposure time, the expression level of *TuGSTm2* was significantly up-regulated, and the relative expression level reached the maximum at 39 °C for 6 h. When exposed to 42 °C, the relative expression level of *TuGSTm2* was significantly up-regulated; it increased with the extension of exposure time, and reached the maximum at 42 °C for 6 h (Figure 4B).

When exposed to 36 °C, the relative expression level of the *TuGSTm3* gene was significantly up-regulated and reached the maximum after 36 °C for 2 h. With the extension of exposure time, the relative expression level of the *TuGSTm3* gene was significantly down-regulated and reached the lowest level compared with the control group at 36 °C for 6 h. When exposed to 39 °C, the relative expression level of the *TuGSTm3* gene was significantly up-regulated. The general trend showed that the relative expression level increased first and then decreased, and the relative expression level reached the maximum at 39 °C for 4 h and the minimum at 39 °C for 6 h. When exposed to 42 °C, the expression level of *TuGSTm3* was significantly up-regulated; it increased with the extension of exposure time, and reached the maximum at 6 h (Figure 4C).

When exposed to 36 °C, the relative expression level of *TuGSTo* was significantly up-regulated and reached the maximum at 36 °C for 2 h, but had no significant difference at 36 °C for 4 h, and was significantly up-regulated again at 36 °C for 6 h. When exposed to 39 °C, the relative expression level of *TuGSTo* was significantly up-regulated; it increased with the extension of exposure time, and reached the maximum at 6 h. When exposed to 42 °C, the relative expression level of *TuGSTo* was significantly up-regulated, and the general trend showed that the expression level of *TuGSTo* increased first, then decreased and then increased again, reaching the maximum at 2 h exposure and the minimum at 4 h exposure (Figure 4D).

When exposed to 36 °C, the relative expression level of *TuGSTd1* was significantly down-regulated and reached the minimum at 36 °C for 6 h. At 39 °C, the relative expression level of *TuGSTd1* was significantly up-regulated only after exposure at 39 °C for 6 h. At 42 °C, the relative expression levels of *TuGSTd1* were significantly up-regulated; they increased with the extension of exposure time, and reached the maximum at 42 °C for 6 h (Figure 4E).

When exposed to 36 °C, the relative expression level of *TuGSTd2* was significantly down-regulated, and decreased with the extension of exposure time, reaching the minimum at 36 °C for 6 h. At 39 °C, there was no significant difference in the relative expression level of *TuGSTd2* after 2 h exposure at 39 °C compared with the control group, and the relative expression level of *TuGSTd2* was significantly down-regulated at 39 °C for 4 h, and only significantly up-regulated and reached the maximum at 39 °C for 6 h. At 42 °C, the relative expression levels of *TuGSTd2* were significantly up-regulated; they increased with the extension of exposure time, and reached the maximum at 42 °C for 6 h (Figure 4F).

## 4. Discussion

*T. urticae* is a notorious worldwide pest known for its wide range of hosts [29], rapid reproduction rate [30], and strong adaptability to chemical pesticides and adverse environments [31]. Especially in the high-temperature summer season, the reproduction rate is higher and the harm is more serious [32]. As a multifunctional protein, GST plays an indispensable role in vivo [33]. GSTs were initially of concern due to their involvement in insecticide resistance and phytochemical detoxification [34], but in recent years, as more and more GST genes have been identified, other functions have been reported including having antioxidant activities [35], promoting inflammatory responses [36], participating in the regulation of immune responses and cell signaling cascades [37], regulating cholesterol transport and/or metabolism involved in ecdysteroid biosynthesis [38], host feeding adaptive conversion processes [39,40], and odor molecular degradation [41], and participating in insect reproductive physiological processes [42]. The aim of this study was to explore the molecular mechanism of GSTs in response to heat stress in *T. urticae*.

In this study, we screened three Mu family GSTs, one Omega family GST, and two Delta family GSTs from the transcriptome of *T. urticae* acquired after heat stress, and preliminarily determined that these six GST genes can reduce the sensitivity of *T. urticae* to high temperature. According to the whole genome information of *T. urticae*, there are 31 GSTs in total in *T. urticae*, all of which belong to cytoplasmic GSTs, including four subfamilies such as Delta, Mu, Omega, and Zeta [25]. According to substrate specificity and immune characteristics, mammalian cytoplasmic GSTs can be divided into Alpha, Mu, Pi, Theta, Zeta, Omega, Sigma, and Kappa families [43]. Higher plant cytoplasmic GSTs can be divided into fourteen families. They are Tau, Phi, Zeta, Theta, TCHQ, Iota (GSTIs), Hemerythrin (GSTHs), DHARs, Lambda (GSTLs), GHRs, mPGES-2s, metaxin, EF1B, and Ure2p [44]. In addition to the four common families of Theta, Zeta, Omega, and Sigma, two unique families of Delta and Epsilon have been found in insects [45]. It is generally believed that proteins whose primary structural sequence similarity is greater than 40% are considered to belong to the same family [43].

The specific GST genes up-regulated during heat stress may vary depending on the organism and its specific physiological and environmental conditions [46]. Different GST isoforms may have distinct substrate specificities and cellular localization [47], allowing them to target different ROS or detoxify specific compounds produced during heat stress. Therefore, we supposed that the reason for the upregulation of these six genes after short-term heat shock is that they share the same specific substrate.

The structure of GSTs in the same family is similar. The closer the phylogenetic relationship is, the more similar the structure is, and the number and location of substrate-binding sites are similar. The phylogenetic analysis of the six GST genes in *T. urticae* showed that *TuGSTm2* and *TuGSTm3* had the closest phylogenetic relationship, *TuGSTd1* and *TuGSTd2* had the closest phylogenetic relationship, and *TuGSTo* was a separate branch. We predicted the tertiary structure of the six GSTs and the number and location of G-sites and H-sites. Although the amino acids at each active site of GSTs of the same family were different, the locations and number of active sites bound to the substrate were similar. For example, the G-site active sites of *TuGSTm1*, *TuGSTm2*, and *TuGSTm3* are all in the 7th, 8th, 46th, 50th, 59th, 60th, 72nd, and 73rd places, with eight G-sites and five H-sites. *TuGSTd1* and *TuGSTd2* each have nine H-sites.

**Figure 5:**
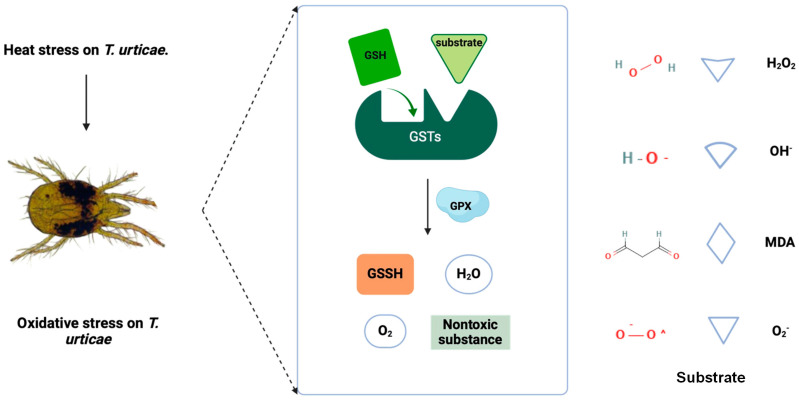
The role of GST in response to short-term heat stress in *T. urticae*. Two domains on the subunits together constitute the active center of GSTs: an N-terminal GSH-binding domain (G-site) and a C-terminal hydrophobic substrate-binding domain (H-site). The harmful substances produced by oxidative stress caused by heat stress mainly include ROS and lipid peroxides. GPX catalyzes the binding of GST and substrate, completes the metabolism of the substrate, and catalyzes GSH to become GSSH in the case of GSH as an electron donor (GPX: Glutathione peroxidase; GSSH: Glutathione(Oxidized); GSH: Glutathione).

The relative expression levels of the six GST genes increased significantly after exposure to heat stress, especially at 42 °C. Among them, the relative expression levels of *TuGSTm1*, *TuGSTm2*, *TuGSTd1*, and *TuGSTd2* reached the highest level after 6 h exposure at 42 °C, and the relative expression levels of *TuGSTm3* and *TuGSTo* reached the highest level after 2 h exposure at 42 °C. We supposed that the potential reason for the disparity between the different recombinant GSTs in the time taken to reach the highest expression is the different affinity for the substrate. Excessive temperatures can cause oxidative stress in organisms [48]. Our previous results showed that short-term heat shock could cause oxidative stress in *T. urticae*, and the activities of three antioxidant enzymes (SOD, Prx, and CAT) and total antioxidant capacity (T-AOC) were significantly increased [9]. After short-term heat shock, the relative expression levels of the six GST genes were significantly up-regulated, indicating that the six GST genes were involved in clearing excess ROS generated by heat stress and reducing the sensitivity of the *T. urticae* to high temperature (Figure 5).

## 5. Conclusions

We used comparative transcriptomics to screen out six GST genes whose relative expression levels were up-regulated after short-term heat shock in *T. urticae*. We carried out the detailed description and phylogenetic analysis of these six GST genes, predicting the tertiary structure and active sites (G-sites and H-sites) of these six GSTs. The expression patterns of the six GST genes in response to heat stress were determined, and finally, the role of the six GST genes involved in the response to heat stress was determined.

## Figures and Tables

**Table 1:** Detailed information of GST genes from *T. urticae*.

Gene	ORF	aa	Formula	Molecular Weight (kDa)	Theoretical pI
*TuGSTm1*	672	224	C_1207_H_1829_N_303_O_346_S_6_	26.313	6.00
*TuGSTm2*	696	232	C_1233_H_1856_N_302_O_358_S_3_	26.734	5.01
*TuGSTm3*	675	225	C_1193_H_1846_N_292_O_353_S_7_	26.152	4.71
*TuGSTd1*	657	219	C_1123_H_1752_N_280_O_327_S_4_	24.536	5.82
*TuGSTd2*	642	192	C_1015_H_1575_N_259_O_293_S_11_	22.447	5.49
*TuGSTo*	732	244	C_1290_H_1987_N_307_O_365_S_10_	27.957	6.12

## Data Availability

All data support for this research is included in this article and Supplementary File.

## Supplementary Materials

The following supporting information can be downloaded at: https://www.mdpi.com/article/10.3390/antiox13040442/s1. Tables S1–S4. Table S1. The primers used for cloning of six GSTs genes. Table S2. The primers used for RT-qPCR of six antioxidant genes. Table S3. The FPKM value of six GST genes. Table S4. The source data of expression levels of six GST genes after heat stress.

## References

[1] Shang S.Q., Chang Y., Li W.Z., Wang C.Q., Nie P.C. (2022). Effects of B-azolemiteacrylic on life-history traits and demographic parameters of two-spotted spider mite, *Tetranychus urticae* (Acari: Tetranychidae). Exp. Appl. Acarol..

[2] Sousa V.C., Zélé F., Rodrigues L.R., Godinho D.P., de la Masselière M.C., Magalhães S. (2019). Rapid host-plant adaptation in the herbivorous spider mite *Tetranychus urticae* occurs at low cost. Curr. Opin. Insect Sci..

[3] Wermelinger B., Oertli J.J., Baumgärtner J. (1991). Environmental factors affecting the life-tables of *Tetranychus urticae* (Acari: Tetranychidae) III. Host-plant nutrition. Exp. Appl. Acarol..

[4] Savi P.J., Gonsaga R.F., de Matos S.T.S., Braz L.T., de Moraes G.J., de Andrade D.J. (2021). Performance of *Tetranychus urticae* (Acari: Tetranychidae) on three hop cultivars (*Humulus lupulus*). Exp Appl Acarol..

[5] Choi Y.S., Kim M.J., Baek S. (2022). Within-Plant Distribution of Two-Spotted Spider Mites, *Tetranychus urticae* Koch (Acari: Tetranychidae), on Strawberries: Decision of an Optimal Sampling Unit. Insects.

[6] Gerson U., Helle W., Sabelis M.W. (1985). Spider Mites: Their Biology. Natural Enemies and Control.

[7] Tetsuo G., Daisuke M., Gösta N. (2015). Development and reproduction of five *Tetranychus* species (Acari: Tetranychidae): Do they all have the potential to become major pests?. Exp. Appl. Acarol..

[8] Tscholl T., Nachman G., Spangl B., Scalmani I., Walzer A. (2023). Parental exposure to heat waves improves offspring reproductive investment in *Tetranychus urticae* (Acari: Tetranychidae), but not in its predator, *Phytoseiulus persimilis* (Acari: Phytoseiidae). Ecol. Evol..

[9] Nie P.C., Yang R.L., Zhou J.J., Dewer Y., Shang S.Q. (2023). Elucidating the Effect of Temperature Stress on the Protein Content, Total Antioxidant Capacity, and Antioxidant Enzyme Activities in *Tetranychus urticae* (Acari: Tetranychidae). Insects.

[10] Forouzanfar M.H., Afshin A., Alexander L.T., Anderson H.R., Bhutta Z.A., Biryukov S., Brauer M., Burnett R., Cercy K., Charlson F.J. (2016). Global, regional, and national comparative risk assessment of 79 behavioural, environmental and occupational, and metabolic risks or clusters of risks, 1990–2015: A systematic analysis for the Global Burden of Disease Study 2015. Lancet.

[11] Ozougwu J.C. (2016). The role of reactive oxygen species and antioxidants in oxidative stress. Int. J. Res..

[12] Aruoma O.I. (1998). Free radicals, oxidative stress, and antioxidants in human health and disease. J. Am. Oil Chem. Soc..

[13] Fairley L.H., Das S., Dharwal V., Amorim N., Hegarty K.J., Wadhwa R., Mounika G., Hansbro P.M. (2023). Mitochondria-Targeted Antioxidants as a Therapeutic Strategy for Chronic Obstructive Pulmonary Disease. Antioxidants.

[14] Scandalios J.G. (2002). The rise of ROS. Trends Biochem. Sci..

[15] Tuzet A., Rahantaniaina M.S., Noctor G. (2019). Analyzing the function of catalase and the ascorbate–glutathione pathway in H2O2 processing: Insights from an experimentally constrained kinetic model. Antioxid. Redox Signal..

[16] Zeng J., Ding C., Chen L., Yang B., Li M., Wang X., Su F., Liu C., Huang Y. (2023). Multienzyme-Mimicking Au@Cu_2_O with Complete Antioxidant Capacity for Reactive Oxygen Species Scavenging. ACS Appl. Mater. Interfaces.

[17] Board P.G., Menon D. (2013). Glutathione transferases, regulators of cellular metabolism and physiology. Biochim. Biophys. Acta BBA-Gen. Subj..

[18] Fragoso D.B., Guedes R.N.C., Rezende S.T. (2003). Glutathione S-transferase detoxification as a potential pyrethroid resistance mechanism in the maize weevil *Sitophilus zeamais*. Entomol. Exp. Appl..

[19] Heidel-Fischer H.M., Vogel H. (2015). Molecular mechanisms of insect adaptation to plant secondary compounds. Curr. Opin. Insect Sci..

[20] Zhang Y., Yan H., Lu W., Li Y., Guo X., Xu B. (2013). A novel Omega-class glutathione S-transferase gene in *Apis cerana cerana*: Molecular characterisation of GSTO2 and its protective effects in oxidative stress. Cell Stress Chaperones.

[21] BK S.K., Moural T., Zhu F. (2022). Functional and structural diversity of insect glutathione S-transferases in xenobiotic adaptation. Int. J. Biol. Sci..

[22] Simon J.Y. (1996). Insect glutathione S-transferases. Zool. Stud..

[23] Giorgio M., Trinei M., Migliaccio E., Pelicci P.G. (2007). Hydrogen peroxide: A metabolic by-product or a common mediator of ageing signals?. Nat. Rev. Mol. Cell Biol..

[24] Liu S., Liu F., Jia H., Yan Y., Wang H., Guo X., Xu B. (2016). A glutathione S-transferase gene associated with antioxidant properties isolated from *Apis cerana cerana*. Sci. Nat..

[25] Grbić M., Van Leeuwen T., Clark R.M., Rombauts S., Rouzé P., Grbić V., Osborne E.J., Dermauw W., Ngoc P.C., Ortego F. (2011). The genome of *Tetranychus urticae* reveals herbivorous pest adaptations. Nature.

[26] Jumper J., Evans R., Pritzel A., Green T., Figurnov M., Ronneberger O., Tunyasuvunakool K., Bates R., Žídek A., Potapenko A. (2021). Highly accurate protein structure prediction with AlphaFold. Nature.

[27] Li W.Z., Kang W.J., Zhou J.J., Shang S.Q., Shi S.L. (2023). The antennal transcriptome analysis and characterizations of odorant-binding proteins in *Megachile saussurei* (Hymenoptera, Megachilidae). BMC Genom..

[28] Livak K.J., Schmittgen T.D. (2001). Analysis of relative gene expression data using real-time quantitative PCR and the 2 (-Delta Delta C(T)) Method. Methods.

[29] Van den Boom C.E.M., Van Beek T.A., Dicke M. (2003). Differences among plant species in acceptance by the spider mite *Tetranychus urticae* Koch. J. Appl. Entomol..

[30] Praslička J., Huszár J. (2004). Influence of temperature and host plants on the development and fecundity of the spider mite *Tetranychus urticae* (Acarina: Tetranychidae). Plant Prot. Sci..

[31] Van Leeuwen T., Vontas J., Tsagkarakou A., Tirry L. (2009). Mechanisms of acaricide resistance in the two-spotted spider mite *Tetranychus urticae*. Biorational Control of Arthropod Pests: Application and Resistance Management.

[32] Bounfour M., Tanigoshi L.K. (2001). Effect of temperature on development and demographic parameters of *Tetranychus urticae* and *Eotetranychus carpini borealis* (Acari: Tetranychidae). Ann. Entomol..

[33] Friedman R. (2011). Genomic organization of the glutathione S-transferase family in insects. Mol. Phylogenet. Evol..

[34] Sun X.Q., Zhang M.X., Yu J.Y., Jin Y., Ling B., Du J.P., Li G.H., Qin Q.M., Cai Q.N. (2013). Glutathione S-transferase of brown planthoppers (*Nilaparvata lugens*) is essential for their adaptation to gramine-containing host plants. PLoS ONE.

[35] Hayes J.D., Strange R.C. (1995). Potential contribution of the glutathione S-transferase supergene family to resistance to oxidative stress. Free Radic. Res..

[36] Hughes M.M., Hooftman A., Angiari S., Tummala P., Zaslona Z., Runtsch M.C., McGettrick A.F., Sutton C.E., Diskin C., Rooke M. (2019). Glutathione Transferase Omega-1 Regulates NLRP3 Inflammasome Activation through NEK7 Deglutathionylation. Cell Rep..

[37] Kim K., Kim S.H., Kim J., Kim H., Yim J. (2012). Glutathione s-transferase omega 1 activity is sufficient to suppress neurodegeneration in a Drosophila model of Parkinson disease. J. Biol. Chem..

[38] Škerlová J., Lindström H., Gonis E., Sjödin B., Neiers F., Stenmark P., Mannervik B. (2020). Structure and steroid isomerase activity of Drosophila glutathione transferase E14 essential for ecdysteroid biosynthesis. FEBS Lett..

[39] Saisawang C., Wongsantichon J., Ketterman A.J. (2012). A preliminary characterization of the cytosolic glutathione transferase proteome from *Drosophila melanogaster*. Biochem. J..

[40] Zou X., Xu Z., Zou H., Liu J., Chen S., Feng Q., Zheng S. (2016). Glutathione S-transferase SlGSTE1 in *Spodoptera litura* may be associated with feeding adaptation of host plants. Insect Biochem. Mol. Biol..

[41] Huang X., Fan D., Liu L., Feng J. (2017). Identification and Characterization of Glutathione S-Transferase Genes in the Antennae of Codling Moth (Lepidoptera: Tortricidae). Ann. Entomol..

[42] Ma M., Zhang Y.X., Chen D., Smagghe G., Wang J.J., Wei D. (2022). Functional characterization of a glutathione S-transferase gene GSTe10 that contributes to ovarian development in *Bactrocera dorsalis* (Hendel). Entomol. Gen..

[43] Sheehan D., Meade G., Foley V.M., Dowd C.A. (2001). Structure, function and evolution of glutathione transferases: Implications for classification of non-mammalian members of an ancient enzyme superfamily. Biochem. J..

[44] Vaish S., Gupta D., Mehrotra R., Mehrotra S., Basantani M.K. (2020). Glutathione S-transferase: A versatile protein family. 3 Biotech..

[45] Enayati A.A., Ranson H., Hemingway J. (2005). Insect glutathione transferases and insecticide resistance. Insect Mol. Biol..

[46] Rezaei M.K., Shobbar Z.S., Shahbazi M., Abedini R., Zare S. (2013). Glutathione S-transferase (GST) family in barley: Identification of members, enzyme activity, and gene expression pattern. J. Plant Physiol..

[47] Raza H. (2011). Dual localization of glutathione S-transferase in the cytosol and mitochondria: Implications in oxidative stress, toxicity and disease. FEBS J..

[48] Zhu T., Li W., Xue H., Dong S., Wang J., Shang S., Dewer Y. (2023). Selection, Identification, and Transcript Expression Analysis of Antioxidant Enzyme Genes in *Neoseiulus barkeri* after Short-Term Heat Stress. Antioxidants.

