# Supplementary material for: Glutathione S-Transferase Genes Involved in Response to Short-Term Heat Stress in Tetranychus urticae (Koch)
Source: Antioxidants (Basel). 2024 Apr 8;13(4):442. doi: 10.3390/antiox13040442 (PMC11047457; doi:10.3390/antiox13040442)
Supplement: Supplementary file 1 [file antioxidants-13-00442-s001.zip › Table S2.pdf]

Table S2. The primers used for RT-qPCR of six GST genes.

Gene ID	Primer Name	Sequence (5'-3')
XM_015925628.2	*TuGSTm1*-F	TGGCTCCTGTTCTTGGCTAT
XM_015925628.2	*TuGSTm1*-R	TCCGGAGCTGGTCCATAGTT
XM_015927346.2	*TuGSTm2*-F	TCGGTTCGGAAGAAGCTGAG
XM_015927346.2	*TuGSTm2*-R	GACGCTTCCTCACCAGCAAA
XM_015927509.2	*TuGSTm3*-F	GGCTCATACAGGCCAAGAGT
XM_015927509.2	*TuGSTm3*-R	TAGCGGATGATAGCGACCGT
XM_015932051.2	*TuGSTo*-F	GGAGCATTTGCAGGCAATCA
XM_015932051.2	*TuGSTo*-R	AGAACTCGCCTGACGAATGG
XM_015936066.2	*TuGSTd1*-F	CAATCGATGGCTGCATTGGG
XM_015936066.2	*TuGSTd1*-R	CGATTCGAGTGAGGCTGGTT
XM_015937313.2	*TuGSTd2*-F	AGTCCACCTTGTCGAACAGT
XM_015937313.2	*TuGSTd2*-R	TCAACCAAAGTGGGAACCACA
JN881327.1	*α-tubulin*-F	TCTTGTCCCCTACCCTCGTA
JN881327.1	*α-tubulin*-R	TTTCCATGTCGAGGGTCACA
