# Supplementary material for: Glutathione S-Transferase Genes Involved in Response to Short-Term Heat Stress in Tetranychus urticae (Koch)
Source: Antioxidants (Basel). 2024 Apr 8;13(4):442. doi: 10.3390/antiox13040442 (PMC11047457; doi:10.3390/antiox13040442)
Supplement: Supplementary file 1 [file antioxidants-13-00442-s001.zip › Table S3.pdf]

Table S3. The FPKM value of six GST genes.

Gene Name	Control 1	Control 2	Control 3	Control 4	Treat 1	Treat 2	Treat 3	Treat 4
TuGSTm1	107.03	120.47	101.73	122.816	179.259	162.455	177.664	156.223
TuGSTm2	9.86	7.725	10.715	6.261	14.253	11.566	12.069	10.01
TuGSTm3	10.468	9.706	9.727	12.128	12.856	12.439	14.226	14.662
TuGSTo	42.179	46.772	40.462	48.41	73.19	80.259	82.111	73.954
TuGSTd1	32.077	29.769	22.684	32.411	40.634	32.097	38.255	35.31
TuGSTd2	5.984	6.984	6.221	8.117	11.656	12.778	14.591	11.935
